# CircHmbox1 Targeting miRNA-1247-5p Is Involved in the Regulation of Bone Metabolism by TNF-α in Postmenopausal Osteoporosis

**DOI:** 10.3389/fcell.2020.594785

**Published:** 2020-12-23

**Authors:** Zhuochao Liu, Changwei Li, Ping Huang, Fangqiong Hu, Min Jiang, Xing Xu, Bin Li, Lianfu Deng, Tianwen Ye, Lei Guo

**Affiliations:** ^1^Department of Orthopedics, Shanghai Key Laboratory for Prevention and Treatment of Bone and Joint Diseases, Shanghai Institute of Traumatology and Orthopedics, Ruijin Hospital, Shanghai Jiao Tong University School of Medicine, Shanghai, China; ^2^Department of Orthopedic Surgery, Changzheng Hospital, Second Military Medical University, Shanghai, China

**Keywords:** postmenopausal osteoporosis, circHmbox1, microRNA-1247-5p, TNF-α, B cell lymphoma 6

## Abstract

Tumor necrosis factor-alpha (TNF-α) promotes osteoclasts differentiation to enhance bone resorption and inhibits osteoblasts differentiation to impair bone formation, which plays a central role in the development of postmenopausal osteoporosis (PMOP). Recent studies implicated an important role of circular RNAs (circRNAs) in osteoporosis. The purpose of this study is to investigate whether circRNAs might be implicated in TNF-α-regulated osteoclasts differentiation and osteoblasts differentiation in PMOP. QRT-PCR was applied to detect expression of circRNA-circHmbox1 and miR-1247-5p in TNF-α-induced osteoclasts differentiation. Western blot, TRAP staining, alkaline phosphatase staining, alizarin red S staining, transwell and cell transfection were conducted to confirm that TNF-α inhibited osteoblasts differentiation by exosomal with low circHmbox1 expression from osteoclasts. Bioinformatics analysis and luciferase reporter revealed the mechanisms of the circHmbox1/miR-1247-5p/B cell lymphoma 6 (Bcl6) interaction. In this study, we found that the level of circRNA-circHmbox1 was obviously reduced in TNF-α-induced osteoclast formation *in vivo* and *in vitro*. CircHmbox1 could inhibit RANKL-induced osteoclasts differentiation primarily through binding to microRNA-1247-5p. TNF-α decreased osteoblasts differentiation by exosomal with low circHmbox1 expression from osteoclasts. Mechanistic studies showed that microRNA-1247-5p regulated osteoclasts differentiation and osteoblasts differentiation by targeting Bcl6, which was confirmed to play opposite roles in osteoblasts differentiation and osteoclasts differentiation. Our results provide evidence that circHmbox1-targeting miR-1247-5p is involved in the regulation of bone metabolisms by TNF-α in PMOP.

## Introduction

Postmenopausal osteoporosis (PMOP) is caused by the imbalance between bone formation by osteoblasts and bone resorption by osteoclasts after estrogen deficiency, which is the most common metabolic bone disease. Many studies demonstrated that the level of inflammation factor tumor necrosis factor alpha (TNF-α) was obviously increased in postmenopausal women, which exerted important regulatory effects on bone turnover in estrogen deficiency-induced osteoporosis (Kawai et al., 2012; Osta et al., 2014). TNF-α has been confirmed to affect bone metabolisms through promoting osteoclasts differentiation and inhibiting osteoblasts differentiation (Zhao, 2017). However, the exact mechanisms by which TNF-α regulates the differentiation of osteoblasts and osteoclasts are not fully elucidated. Thus, exploring the molecular mechanisms that TNF-α regulates osteoblasts and osteoclasts differentiation is critical for researching and treating PMOP.

Circular RNAs (circRNAs) are a novel type of noncoding RNAs, characterized by their covalently closed loop structures with 3′ heads and 5′ tails bound together (Ebbesen et al., 2016; Haddad and Lorenzen, 2019). CircRNAs could bind to microRNAs (miRNAs) to regulate gene expression at the transcriptional or post-transcriptional level. MiRNAs are involved in cell proliferation, apoptosis, and cancer (Khan et al., 2019), which inhibit translational and/or mRNA degradation of target mRNAs through the inhibitory engagement of complimentary “seed sequences” within the 3′-untranslational region (3′-UTR) of target mRNAs (Verduci et al., 2019). Growing evidences have confirmed that miRNAs were involved in the regulation of bone turnover by TNF-α in estrogen deficiency-induced osteoporosis (Maeda et al., 2017). However, it is unclear whether circRNAs are also participated in TNF-α-regulated bone metabolism in PMOP.

Exosomes are membrane-enclosed vesicles (30–50 nm), which are secreted by a multitude of cell types and can transfer cargo (proteins, nucleic acids and lipids) to influence the physiological behavior of recipient cells (Wu et al., 2019). Exosomes play an important role in the intercellular communication and various biological processes, such as in an endocrine manner to regulate a neighboring cell or distant cells after entering the blood stream (Wu et al., 2019). Many studies have demonstrated that nucleic acids-enriched exosomes were involved in the communication between osteoblasts and osteoclasts and were used as potential biomarker for bone metabolic diseases (Xie et al., 2017). However, it is unclear whether nucleic acids-enriched exosomes are involved in the regulation of bone metabolisms by TNF-α in PMOP.

In this study, we found that the level of circRNA mmu_circ_0000549 (mmu_circ_0000549) was obviously decreased in the induction of osteoclasts differentiation by TNF-α *in vivo* and *in vitro*. Mmu_circ_0000549 is located at chr14:65444437-65447505 with 3068 lengths in gene symbol Hmbox1. Thus, we nominate it as circHmbox1. CircHmbox1 was further confirmed to decrease osteoclasts differentiation primarily through inhibition of miR-1247-5p targeting B cell lymphoma 6 (Bcl6). Furthermore, TNF-α stimulated osteoclasts to produce the exosomal with low circHmbox1 expression, which could inhibit osteoblasts differentiation through miR-1247-5p targeting Bcl6. Taken together, these results showed that circHmbox1-targeting miR-1247-5p was involved in the regulation of bone metabolisms by TNF-α in PMOP.

## Materials and Methods

### Animal Models

All animal care and experimental procedures were approved by the Shanghai Jiao Tong University Animal Study Committee and were carried out according to the guide for the laboratory animal’s use (Ethics No. 20190321564). Animal models were made as previously described (Du et al., 2018). Eight-week-old female C57BL/6J mice were randomly divided into five groups: SHAM group, ovariectomized (OVX) group, OVX+ TNF-neutralization (anti-TNF-α group), circHmbox-carrying chitosan (circHmbox-CH) group and empty vector-carrying chitosan (Mock) group. The mice in the SHAM group only underwent surgical incision exposing bilateral ovaries. The bilateral ovaries of mice were removed in OVX group. In OVX+anti-TNF-α group, mice were injected with 100 mg/kg-body weight of anti-TNF-α (R&D Systems, MN, United States) via a tail vein injection two times per week. In circHmbox-CH group and mock group, mice were delivered nanoparticles by intravenous injections at 5 mg per mouse twice a week. Chitosan was first dissolved in 0.25% acetic acid solution to make the final concentration at 2 mg/ml. Subsequently, the circHmbox plasmid was mixed with 0.25% sodium tripolyphosphate (TPP) solution to a concentration of 1 mg/ml. Finally, the chitosan solution and TPP mixed solution was mixed in the ratio of 1:6 for intravenous injection. After eight weeks, the femurs and tibia were acquired from different groups for micro computed tomography (micro-CT) scan, tartrate resistant acid phosphatase (TRAP) staining (Sigma-Aldrich, St Louis, MO, United States), hematoxylin eosin (HE) staining and cell culture. In addition, the mice were intraperitoneally injected with 30 mg/kg body weight of calcein (Sigma-Aldrich, St Louis, MO, United States) in 10 days and 3 days before the execution.

### Skeletal Phenotyping

Bone mass, bone density and trabecular microarchitecture were measured by micro-CT (GE Locus SP) in the distal end of intact femurs of SHAM, OVX and OVX+anti-TNF-α mice. The threshold, 80∼255, was chosen to segment out bone. That resolution for micro-CT imaging was 10 μm. And the total number of images we acquired for 3D reconstruction was 100. Bone mineral density (BMD), trabecular thickness (Tb.Th), bone volume against tissue volume (BV/TV), and trabecular number (Tb.N) were calculated from these data.

### Histological Analysis

Samples were fixed with 4% paraformaldehyde and then were decalcificated with EDTA-buffered saline solution (pH 7.4, 0.25 M). The entire decalcification process continued for 3 weeks. And then the samples were paraffin-embedded and sectioned, followed by cutting longitudinally to obtain 10 μm sections. then the sections were stained with TRAP staining and HE staining. The sections were imaged by a Zeiss microscope (Carl Zeiss, Oberkochen, Germany).

### Cell Culture

Primary bone marrow macrophages cells (BMMs) were acquired from the long bones in 6-week-old C57BL/6J mice. BMMs were cultured in medium with 50 ng/ml macrophage-colony stimulating factor (M-CSF) for 3 days to form osteoclast precursors (pre-osteoclasts). Then pre-osteoclasts were induced with 50 ng/ml receptor activator of nuclear factorκ-B ligand (RANKL) (Peprotech, Rocky Hill, NJ) and 30 ng/ml M-CSF (Peprotech, Rocky Hill, NJ) for 7 days to form mature osteoclasts as previous description (Guo et al., 2018). Raw264.7 cells were cultured in complete alpha-minimal essential media (α-MEM) medium with 50 ng/ml RANKL for 7 days to acquire mature osteoclasts. Primary osteoblast precursor cells were acquired from neonatal murine calvaria as previous description (Kang et al., 2016). The primary osteoblast precursor cells were induced with 4 mmol/L beta-glycerophosphate and 50 μg/ml ascorbic acid (Sigma-Aldrich, St Louis, MI, United States) for 7 days to form mature osteoblasts.

### Co-culture

Osteoclasts and osteoblasts were co-cultured in a transwell system with a 0.4-μm pore polyethylene terephthalate (PET) membrane as previously reported (Sun et al., 2016). The RAW264.7 cells were treated with or without 10 ng/ml TNF-α under 50 ng/ml RANKL for 7 days. Osteoblast precursor cells from the calvarial bone of newborn mice were induced with osteogenic medium for 7 days. Afterwards, the osteoblasts were co-cultured with mature osteoclasts from different treatment groups in α-MEM medium with exosome-depleted fetal bovine serum (FBS) for 2 days.

### Cell Transfection

The overexpression plasmid vector for mouse circHmbox1 and mouse *Bcl6* gene were created by Genechem (Shanghai, China). Small interfering RNAs (siRNAs) targeting the back-splice junction of circHmbox1 (si-circHmbox1) were designed and synthesized by HanBio (Shanghai, China). MiR-1247-5p mimic, miR-NC, anti-microRNA oligonucleotides (AMO)-1247-5p and AMO-NC were synthesized by Genechem (Shanghai, China). Pre-osteoclasts were cultured in complete α-MEM medium with 30 ng/mL M-CSF and 50 ng/mL RANKL for 24 h before transfection. When pre-osteoclasts reached approximately 70% confluence, cells were transfected with miR-1247-5p, AMO-1247-5p or plasmid DNA with Lipofectamine 3000 reagent (Invitrogen, Paisley, United Kingdom).

### Exosome Isolation and Labeling

Exosomes were acquired by differential centrifugations as previously described (Sun et al., 2016). Exosomes were pelleted and the pellets were resuspended in PBS. The fluorescent dye 3, 3′-dioctadecyloxacarbocyanine perchlorate (DIO) (Invitrogen Molecular Probes, Carlsbad, CA, United States) was used to label exosomes and cell membrane.

### TRAP and Rhodamine Phalloidin Staining

The osteoclasts were fixed with 4% paraformaldehyde, followed by incubation in 0.1% Triton X for 5 min. The solution was then replaced with rhodamine phalloidin solution (Sigma-Aldrich, St Louis, MO, United States) for 30 min. Using confocal laser scanning microscopy to test the fluorescence signal (Carl Zeiss, Oberkochen, Germany). The number of osteoclasts with a fluorescent ring was counted. For TRAP staining, cells were incubated in TRAP solution for 30 min at 37°C. The number of cells with 3 or more nuclei was counted.

### RNA Extraction and Quantitative Real-Time PCR (qRT-PCR)

QRT-PCR assay was accomplished as previously described (Sang et al., 2016). The mouse primer sequences for *TRAP (NM_011611), ctsk* (*NM_007802*), *NFATc1 (NM_001164112), Runx2 (NM_001146038), Osterix (NM_130458), β-actin* (*NM_ 007393*), *mmu_circ_0000150 (NM_001033258), mmu_circ_ 0001209 (NM_026079), mmu_circ_0000722 (NM_001085355), mmu_circ_0001660 (NM_053171), mmu_circ_0001667 (NM_02 6032), mmu_circ_0001867 (NM_009481), mmu_circ_0001081 (NM_001199188), mmu_circ_0000320 (NM_001080925), mmu_ circ_0000027 (NM_173870), mmu-miR-1247-5p (MIMAT001 4800), mmu-miR-17-3p (MIMAT0000650), mmu-miR-200c-5p (MIMAT0004663), mmu-miR-222-3p (MIMAT0000670), mmu-miR-27a-3p (MIMAT0000537), mmu-miR-541-5p (MIMAT00 03170), mmu-miR-666-3p (MIMAT0004823)*, *mmu-miR-690 (MIMAT0003469)* and *mmu-U6 (NR_138085.1)* were described in Tables 1, 2.

**TABLE 1:** Primers for qRT-PCR analysis.

Gene	Forward Primer	Reverse Primer
*TRAP*	5′-CACTCCCACCCTGAGATTTGT-3′	5′-CATCGTCTGCACGGTTCTG-3′
*ctsk*	5′-GAAGAAGACTCACCAGAAGCAG-3′	5′-TCCAGGTTATGGGCAGAGATT-3′
*nfatc-1*	5′-GACCCGGAGTTCGACTTCG-3′	5′-TGACACTAGGGGACACATAACTG-3′
*Runx2*	5′-ATGCTTCATTCGCCTCACAAA-3′	5′-GCACTCACTGACTCGGTTGG-3′
*Osterix*	5′-ATGGCGTCCTCTCTGCTTG-3′	5′-TGAAAGGTCAGCGTATGGCTT-3′
*circ_0000150*	5′-TCCAAGGCAACACCAACC-3′	5′-TTTGCTCCTCACAATCCTC-3′
*circ_0001209*	5′-AGAACTCTGGCAGGAAAG-3′	5′-CGCCTGGTACTGTGGTGA-3′
*circ_0000722*	5′-CCATTTGCCTTTCTACCTTG-3′	5′-TGGGTATGGGCTGCTCTGC-3′
*circ_0001660*	5′-CCCTGACCTGCCTACATC-3′	5′-GACAGCACATCGTTCACC-3′
*circ_0001667*	5′-CCCAGTGTTAAGTGCTTTC-3′	5′-GGTCAGCAAATGCCCCT-3′
*circ_0001867*	5′-TAAGGGCTGGAATAAAACAT-3′	5′-TGCAATGGTCTCTGCAAG-3′
*circ_0001081*	5′-TCTTTTCTAACCGTTTTCTG-3′	5′-ATGCAGACACCAAAAGGC-3′
*circ_0000320*	5′-CAGTGGATCTCTTCTGGAA-3′	5′-CGATGTGTGCACGAGGA-3′
*circ_0000027*	5′-TCCCTCTCCTTCCTCCCT-3′	5′-GACTTGAACCCCGCGAG-3′
*β-actin*	5′-GGCTGTATTCCCCTCCATCG-3′	5′-CCAGTTGGTAACAATGCCATGT-3′

**TABLE 2:** MicroRNAs primers for qRT-PCR analysis.

MicroRNAs	RT-Primer	Premiers
*miR-1247-5p*	5′-GTCGTATCCAGTGCGTGTCGTGG AGTCGGCAATTGCACTGGATACGACTCCGGGG-3′	F:5′-GGGACCCGTCCCGTTCGTCC-3′ R: 5′-CAGTGCGTGTCGTGGAGT-3′
*miR-17-3p*	5′-GTCGTATCCAGTGCGTGTCGTGG AGTCGGCAATTGCACTGGATACGACCTACAAG-3′	F:5′-GGGACTGCAGTGAGGGCACTTG-3′ R: 5′-CAGTGCGTGTCGTGGAGT-3′
*miR-200c-5p*	5′-GTCGTATCCAGTGCGTGTCGTGG AGTCGGCAATTGCACTGGATACGACCCAAACA-3′	F:5′-GGGCGTCTTACCCAGCAGTGTT-3′ R: 5′-CAGTGCGTGTCGTGGAGT-3′
*miR-222-3p*	5′-GTCGTATCCAGTGCGTGTCGTGG AGTCGGCAATTGCACTGGATACGACACCCAGT-3′	F:5′-GGGAGCTACATCTGGCTACTG-3′ R: 5′-CAGTGCGTGTCGTGGAGT-3′
*miR-27a-3p*	5′-GTCGTATCCAGTGCGTGTCGTGG AGTCGGCAATTGCACTGGATACGACGCGGAAC-3′	F:5′-GGGTTCACAGTGGCTAAGTT-3′ R: 5′-CAGTGCGTGTCGTGGAGT-3′
*miR-541-5p*	5′-GTCGTATCCAGTGCGTGTCGTGG AGTCGGCAATTGCACTGGATACGACAGTGTGA-3′	F:5′-GGGAAGGGATTCTGATGTTGGT-3′ R: 5′-CAGTGCGTGTCGTGGAGT-3′
*miR-666-3p*	5′-GTCGTATCCAGTGCGTGTCGTGG AGTCGGCAATTGCACTGGATACGACAGCAGGC-3′	F:5′-GGGGGCTGCAGCGTGATCGCCT-3′ R: 5′-CAGTGCGTGTCGTGGAGT-3′
*miR-690*	5′-GTCGTATCCAGTGCGTGTCGTGG AGTCGGCAATTGCACTGGATACGACTTTGGTT-3′	F:5′-GGGAAAGGCTAGGCTCACAAC-3′ R: 5′-CAGTGCGTGTCGTGGAGT-3′
*mmu-U6*	5′-CGCTTCACGAATTTGCGTGTCAT-3′	F:5′-CAAAGTGCTTACAGTGCAGGTAG-3′ R: 5′-CTACCTGCACTGTAAGCACTTTG-3′

### Western Blot Analysis

Cells proteins were harvested according to previous reports (Sang et al., 2016). BCA protein assay kit (Pierce Biotechnology, Rockford, IL, United States) was used to determine the concentrations of protein. Equal amounts of protein lysates were resolved by SDS–PAGE, and then transferred to PVDF membranes (Millipore, Bedford, MA, United States). 5% skimmed milk solution was applied to block the membranes for 1 h. Afterwards, the membranes were incubated in primary antibodies: anti-Bcl6, anti-TSG101, anti-CD63, anti-CD81, anti-HSP70 and anti-β-actin (Abcam, Cambridge, United Kingdom) overnight. A horseradish peroxidase-conjugated secondary antibody (Boster, Wuhan, China) was used to detect the primary antibody.

### Luciferase Reporter Assays

To reveal the interaction of circRNA-miRNA, circHmbox1 sequence containing the putative target sites for miR-1247-5p was cloned into the pMIR-REPORT^TM^ reporter vector (Thermo Fisher Scientific Inc., Waltham, MA, United States) downstream to the firefly luciferase (pMIR-circHmbox1). HEK293 cells were transfected with pMIR-circHmbox1 and 100 nM miR-1247-5p mimic or miR-NC with Lipofectamine 3000 reagent. The cells transfected with 50 ng pRL-TK vector were an internal standard. A dual luciferase reporter assay kit (Promega, Madison, WI, United States) was used to test luciferase activities.

### Alkaline Phosphatase (ALP) Staining and Alizarin Red S (ARS) Staining

The osteoblasts were fixed with 4% formaldehyde for 10 min. Next, the cells were stained with ALP reagent (Beyotime Institute of Biotechnology, Shanghai, China) for 30 min at 37°C. The differentiated osteoblasts were dyed with blue violet (Sang et al., 2016). Pre-osteoblasts were cultured in osteogenic differentiation medium for 14 days, followed by ARS staining (Beyotime Institute of Biotechnology, Shanghai, China). The cells were stained with 0.2% ARS solution for 30 min at 37°C. The red color obtained referred to calcium deposits (Sang et al., 2016).

### Statistical Analysis

Statistical analysis was performed using SPSS software (SPSS 11.5, Chicago, IL, United States). Two-tailed unpaired one-way ANOVA was used to compare between more than two groups. Bars are the mean ± SD of *n* biological samples. Results were considered statistically significant if *P ≤ 0.05*.

## Results

### CircHmbox1 (circ_0000549) Expression Is Decreased in the Induction of Osteoclasts Differentiation by TNF-α

**FIGURE 1:**
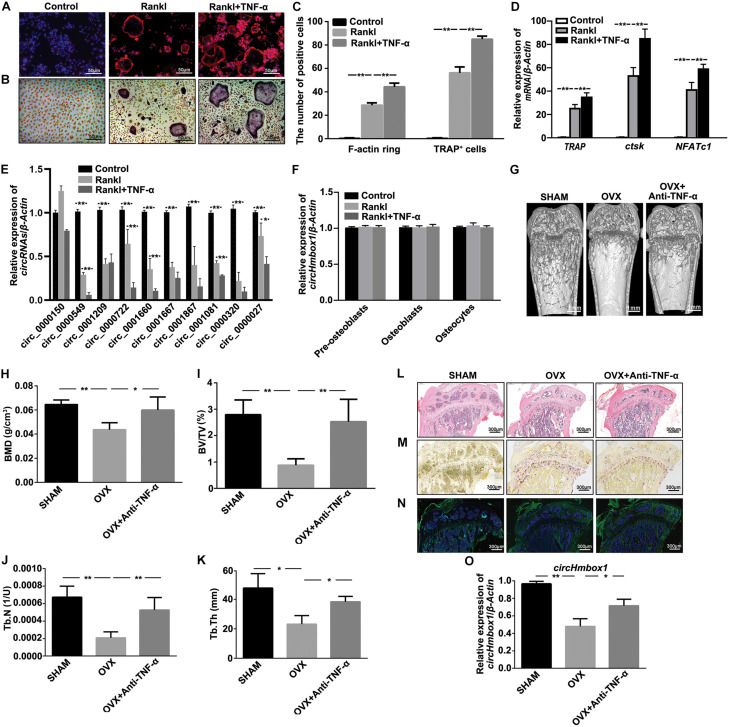
CircHmbox1 (circ_0000549) expression is decreased in the induction of osteoclasts differentiation by TNF-α. **(A)** F-actin ring staining was showed in BMMs treated with or without TNF-α under RANKL and MCSF for 7 days. Scale bars are 50 μm. **(B)** TRAP staining was showed in BMMs treated with or without TNF-α under RANKL and MCSF for 7 days. Scale bars are 50 μm. **(C)** The number and area of F-actin ring and TRAP^+^ cells were counted. *n* = 4, **P < 0.01. **(D)** The expressions of osteoclasts formation specific genes, *TRAP*, *ctsk* and *NFATc1*, were analyzed by qRT-PCR in BMMs treated with or without TNF-α under RANKL and MCSF for 7 days. *n* = 4, **P < 0.01. **(E)** The expressions of circRNAs-associated osteoclastogenesis were analyzed by qRT-PCR in BMMs treated with or without TNF-α under RANKL and M-CSF for 7 days. *n* = 4, *P < 0.05, **P < 0.01. **(F)** The expressions of circHmbox1 were analyzed by qRT-PCR in pre-osteoblasts, osteoblasts and osteocytes. *n* = 3. **(G)** Representative micro-CT three-dimensional reconstructed images from SHAM mice, OVX mice and anti-TNF-α-treated OVX mice. **(H–K)** BMD, BV/TV, Tb.N and Tb.Th in the region of interest were measured. *n* = 6 mice per group. **(L)** HE staining was performed to histologically identify structures of the distal end of intact tibias in SHAM mice, OVX mice and anti-TNF-α-treated OVX mice. **(M)** TRAP staining was showed in the metaphyseal area of tibias bone sections derived from SHAM mice, OVX mice and anti-TNF-α-treated OVX mice. *n* = 4. **(N)** The immunofluorescence staining showed that anti-TNF-α alleviated OVX-inhibited osteoblastic marker osteopontin expression (green fluorescence) in the bone trabecula surface. *n* = 4. **(O)** The expression of circHmbox1 was analyzed by qRT-PCR in osteoclasts from SHAM mice, OVX mice and anti-TNF-α-treated OVX mice. *n* = 4, *P < 0.05, **P < 0.01.

F-actin ring staining showed the clear ring structures in the RANKL group. The number of F-actin ring was significantly increased when BMMs were incubated with 10 ng/ml TNF-α in the presence of RANKL and M-CSF (Figure 1A). TRAP staining showed that the number of mature osteoclasts was significantly increased in RANKL together with TNF-α group compared to RANKL group (Figures 1B,C). In addition, the expressions of osteoclasts marker genes, including *TRAP*, *ctsk* and *NFATc1*, were obviously increased in RANKL-induced osteoclastogenesis, which were further enhanced by TNF-α (Figure 1D). We then tested the expression of circRNAs-associated osteoclastogenesis in TNF-α-induced osteoclasts differentiation. Circ_0000549 (circHmbox1), circ_0001209, circ_0001660, circ_0001667, circ_0001081 and circ_0000320 were found to be downregulated about two-folds, while circ_0000150 was upregulated in RANKL group. All of these circRNAs expression were further decreased in RANKL together with TNF-α group compared to RANKL group. CircHmbox1 was the most significant change during these differentially expressed circRNAs (Figure 1E). We also examined the effect of TNF-α on circHmbox1 expression in pre-osteoblasts, osteoblasts and osteocytes. No significant effects of TNF-α on circHmbox1 expression were observed in these cells (Figure 1F).

The effect of TNF-α on circHmbox1 expression was further examined *in vivo*. Micro-CT analysis revealed that BMD, BV/TV, Tb.N and Tb.Th were decreased in femora from OVX mice compared to SHAM control mice. However, the treatment of anti-TNF-α significantly alleviated OVX-induced osteoporosis (Figures 1G–K). Similar results were observed with HE staining (Figure 1L). Furthermore, TRAP staining revealed that the number of multinucleated osteoclasts was significantly increased in OVX mice compared with the SHAM mice, which was obviously reduced in anti-TNF-α-treated OVX mice (Figure 1M). The immunofluorescence staining showed that anti-TNF-α alleviated OVX-inhibited osteoblastic marker osteopontin expression in the bone trabecula surface, which was evidenced by enhanced green fluorescence of osteopontin in anti-TNF-α-treated OVX mice compared with OVX mice (Figure 1N). These results suggested that anti-TNF-α obviously alleviated OVX-induced osteoporosis *in vivo*. Subsequently, BMMs from different groups of mice were induced with M-CSF and RANKL for 7 days, followed by measurement of *circHmbox1* expression. We found that *circHmbox1* expression was significantly decreased in OVX group, which was alleviated by anti-TNF-α (Figure 1O). These results indicated that TNF-α could decrease circHmbox1 expression *in vivo*, which might be play an important role in OVX-induced osteoporosis.

### CircHmbox1 Is Involved in TNF-α-Induced Osteoclasts Differentiation

**FIGURE 2:**
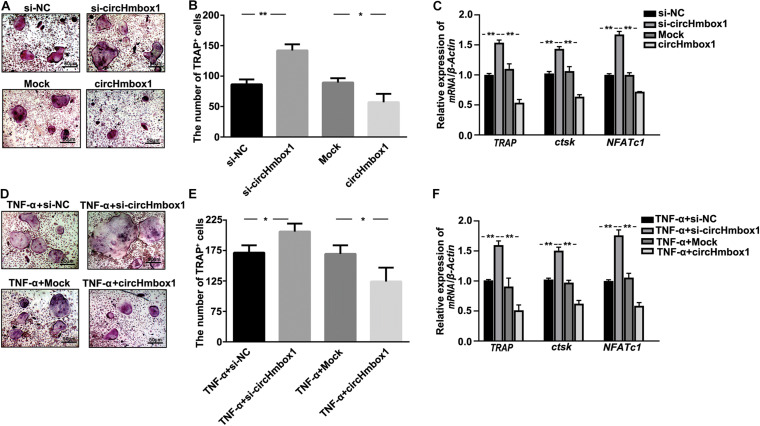
CircHmbox1 is involved in TNF-α-induced osteoclasts differentiation. **(A)** TRAP staining was showed in osteoclasts silencing or overexpressing circHmbox1 under RANKL and M-CSF for 7 days. **(B)** Summarized data showed that silencing of circHmbox1 significantly increased osteoclasts differentiation. However, circHmbox1 significantly inhibited Rankl-induced osteoclastogenesis. *n* = 6, *P < 0.05, **P < 0.01. **(C)** The expressions of osteoclasts formation specific genes, *TRAP*, *ctsk* and *NFATc1*, were analyzed by qRT-PCR in osteoclasts silencing or overexpressing circHmbox1 under RANKL and M-CSF for 7 days. *n* = 4, **P < 0.01. **(D,E)** TRAP staining showed that silencing of circHmbox1 could further enhance the promoting effect of TNF-α on osteoclast differentiation. However, overexpressing of circHmbox1 could attenuate TNF-α-induced osteoclast differentiation. *n* = 6, *P < 0.05. **(F)** The expressions of osteoclasts formation specific genes, *TRAP*, *ctsk* and *NFATc1*, were analyzed by qRT-PCR in osteoclasts overexpressing or silencing circHmbox1 under RANKL, M-CSF and TNF-α for 7 days. *n* = 4, *P < 0.05, **P < 0.01.

To observe the effect of circHmbox1 on osteoclasts differentiation, we decreased and increased the expressions of circHmbox1 in pre-osteoclasts, respectively. TRAP staining showed that silencing of circHmbox1 obviously increased RANKL-induced osteoclast differentiation. In contrast, circHmbox1 could obviously inhibit osteoclast differentiation (Figures 2A,B). QRT-PCR analysis revealed that the levels of the osteoclast-specific genes *TRAP*, *ctsk* and *NFATc1* were higher in osteoclasts silencing of circHmbox1 compared with the cells transfected with si-NC, whereas overexpressing of circHmbox1 significantly inhibited these osteoclast-specific genes expression (Figure 2C). To further examine whether circHmbox1 was involved in TNF-α-induced osteoclasts differentiation, circHmbox1 was silenced or overexpressed in pre-osteoclasts, and then the cells were induced with RANKL and M-CSF for 7 days in the presence of 10 ng/ml TNF-α. TRAP staining showed that silencing of circHmbox1 could further enhance the promoting effect of TNF-α on osteoclast differentiation. However, overexpressing of circHmbox1 could attenuate TNF-α-induced osteoclast differentiation (Figures 2D,E). Similar results were further demonstrated by the measurements of osteoclast-specific genes expression (Figure 2F). Based on the above results, we concluded that circHmbox1 was involved in TNF-α-induced osteoclasts differentiation.

### Exosomal With Low CircHmbox1 Expression From Osteoclasts Treated With TNF-α Inhibits Osteoblast Differentiation

**FIGURE 3:**
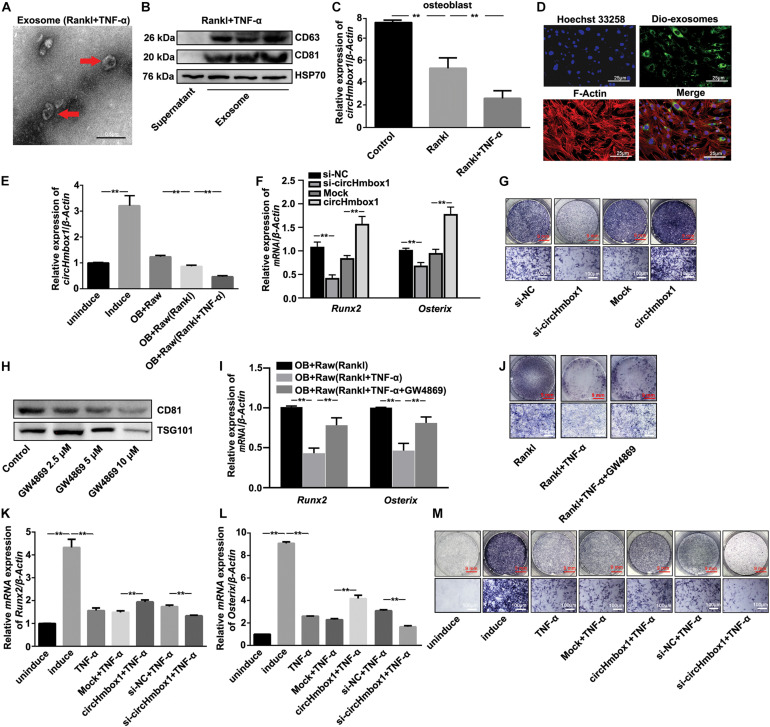
Exosomal circHmbox1 from osteoclasts treated with TNF-α inhibits osteoblast differentiation. **(A)** An electron microscopy image of exosomes. Scale bar = 0.5 μm. **(B)** The expressions of CD63, CD81 and HSP70 in cells lysates and exosomes secreted by RANKL-induced RAW 264.7 cells were analyzed by western blot. **(C)** The expressions of circHmbox1 were analyzed by qRT-PCR in osteoblasts incubated with exosomes from osteoclasts induced with RANKL and/or TNF-α. *n* = 4, **P < 0.01. **(D)** Confocal microscopy images of colocalization of exosomes from RANKL-induced RAW 264.7 cells with osteoblasts. Exosomes were labeled by DiO (green), F-actin were stained with rhodamine-phalloidin (red) and cell nuclei were stained with Hoechst 33342 (blue). Scale bars, 25 μm. **(E)** The expressions of circHmbox1 were analyzed by qRT-PCR in osteoblasts co-cultured with osteoclasts induced with RANKL and/or TNF-α. *n* = 4, **P < 0.01. **(F)** Osteoblasts adding exosomes from osteoclasts increasing or silencing circHmbox1 were cultured in osteogenic differentiation medium for 7 days. QRT-PCR was applied to test the expressions of *Osterix* and *Runx2*. *n* = 4, **P < 0.01. **(G)** Osteoblasts adding exosomes from osteoclasts increasing or silencing circHmbox1 were cultured in osteogenic differentiation medium for 7 days, followed by ALP staining. *n* = 4. **(H)** The expressions of CD81 and TSG101 in cells lysates and exosomes secreted by osteoclasts treated with GW4869 were analyzed by western blot. *n* = 3. **(I)** The expressions of *Runx2* and *Osterix* were analyzed by qRT-PCR in osteoblasts co-cultured with osteoclasts treated with GW4869 in the presence of RANKL and TNF-α. *n* = 3, **P < 0.01. **(J)** Osteoblasts were co-cultured with osteoclasts treated with GW4869 in the presence of RANKL and TNF-α, followed by ALP staining. *n* = 4. **(K–M)** Osteoblasts adding exosomes from osteoclasts increasing or silencing circHmbox1 were cultured in osteogenic differentiation medium for 7 days in presence of TNF-α. QRT-PCR was applied to test the expressions of *Runx2* **(K)** and *Osterix* **(L)**. *n* = 4, **P < 0.01. **(M)** Osteoblasts adding exosomes from osteoclasts increasing or silencing circHmbox1 were cultured in osteogenic differentiation medium for 7 days in presence of TNF-α, followed by ALP staining. *n* = 4.

To characterize the vesicles released in TNF-α-induced osteoclasts differentiation, Raw264.7 cells were induced with RANKL in the presence of TNF-α for 7 days. The medium was then collected and ultracentrifugated to analysis vesicles. Exosomes with a rounded morphology and with a size of 40 to 100 nm in diameter were observed by electron microscopy (Figure 3A). Western blot analysis of exosome markers, including CD63, CD81 and HSP70, in the extracts confirmed the presence of exosomes (Figure 3B). The exosomes from osteoclasts treated with RANKL and/or TNF-α were added into osteoblasts for 2 days, followed by the measurement of circHmbox1 expression. CircHmbox1 expression was significantly decreased in osteoblasts incubated with exosomes from RANKL-induced osteoclasts, which was further decreased by exosomes from osteoclasts treated with RANKL and TNF-α (Figure 3C). To further test whether exosomal circHmbox1 transferred from osteoclasts could affect circHmbox1 expression in osteoblasts, we co-cultured osteoclasts with osteoblasts in a transwell system with a 0.4-μm pore PET membrane. We firstly applied Dio to label the exosomes from osteoclasts. Confocal imaging showed numerous Dio particles were observed within osteoblasts after 48 h co-culture with osteoclasts (Figure 3D). The level of circHmbox1 in osteoblasts co-cultured with Raw264.7 cells treated with RANKL and TNF-α were obviously lower than that in osteoblasts co-cultured with RANKL-induced Raw264.7 cells (Figure 3E). To confirm whether exosomes circHmbox1 from osteoclasts could affect osteoblasts differentiation, we collected exosomes from osteoclasts silencing or increasing circHmbox1 for 48h. We then added these exosomes into osteoblasts cultured in osteogenic differentiation medium, followed by the measurement of osteoblasts marker genes (*Runx2* and *Osterix*) expression by qRT-PCR. The results showed that the expressions of *Runx2* and *Osterix* were decreased in osteoblasts adding exsomes from osteoclasts silencing circHmbox1 when compared with those from osteoclasts transfected with si-NC. However, *Osterix* and *Runx2* expressions were increased in osteoblasts adding exosomes from osteoclasts overexpressing circHmbox1, suggesting that exosomal circHmbox1 from osteoclasts could regulate osteoblasts differentiation (Figure 3F). Similar results were observed with ALP staining (Figure 3G). To further examine that TNF-α inhibited osteoblasts differentiation through the exosomes from osteoclasts. Different concentrations of GW4869, the neutral sphingomyelinase inhibitor, were added into osteoclasts to inhibit exosome secretion (Figure 3H). The results of qRT-PCR and ALP staining showed that inhibition of exosome secretion of osteoclasts with GW4869 obviously alleviated TNF-α-inhibited osteoblasts differentiation co-cultured with osteoclasts, indicated that exosomes from osteoclasts play an important role in the regulation of osteoblasts differentiation by TNF-α (Figures 3I,J). To further confirm whether TNF-α could inhibit osteoblasts differentiation through regulating osteoclasts to secrete exosomes with low circHmbox1 expression, we collected exosomes from osteoclasts increasing or silencing circHmbox1 in the presence of TNF-α. The results of qRT-PCR and ALP staining showed that the osteoblasts differentiation was decreased in osteoblasts adding exosomes from osteoclasts treated with TNF-α, which was obviously alleviated by exosomes from TNF-α-treated osteoclasts overexpressing circHmbox1. In addition, osteoblasts differentiation was further inhibited in osteoblasts treated exosomes from osteoclasts silencing circHmbox1 under TNF-α treatment (Figures 3K–M). These results indicated that TNF-α might inhibit osteoblasts differentiation through promoting osteoclasts to secrete exosomes with low circHmbox1 expression.

### MiR-1247-5p Is a Complementary Targeting miRNA of CircHmbox1

**FIGURE 4:**
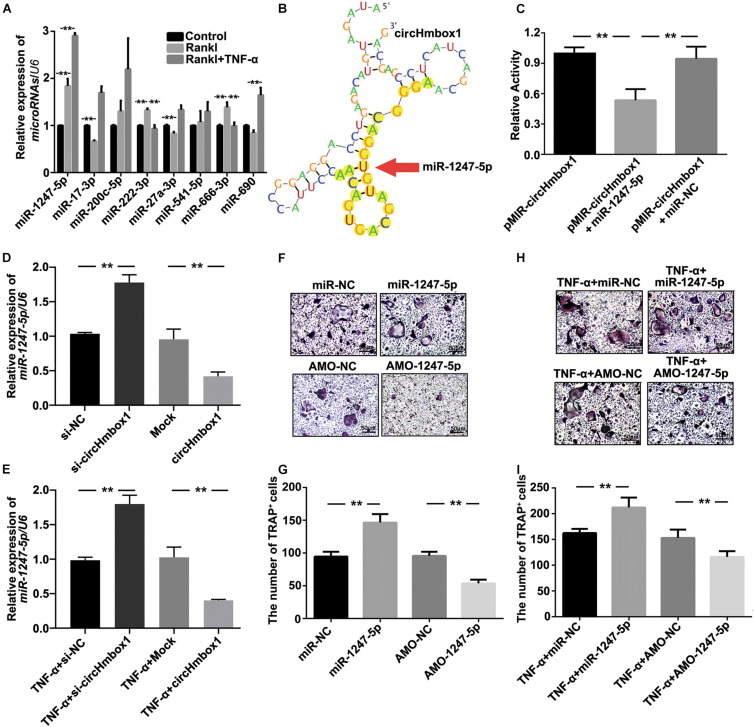
MiR-1247-5p is a complementary targeting miRNA of circHmbox1. **(A)** The expressions of miRNAs binding to circHmbox1 were analyzed by qRT-PCR in TNF-α-induced osteoclasts differentiation. *n* = 3, **P < 0.01. **(B)** Complementation between circHmbox1 and “seed sequence” of miR-1247-5p were showed with yellow highlights. **(C)** Dual luciferase reporter assay displayed the complementary binding of circHmbox1 and miR-1247-5p. *n* = 6, **P < 0.01. **(D)** The expressions of miR-1247-5p were analyzed by qRT-PCR in osteoclasts overexpressing or silencing miR-1247-5p. *n* = 3, **P < 0.01. **(E)** The expressions of miR-1247-5p were analyzed by qRT-PCR in osteoclasts overexpressing or silencing miR-1247-5p in the presence of TNF-α. *n* = 3, **P < 0.01. **(F)** TRAP staining was accomplished in osteoclasts overexpressing/inhibiting miR-1247-5p in the presence of RANKL and M-CSF for 5 days. **(G)** Summarized data showed that miR-1247-5p could promote RANKL-induced osteoclast differentiation. However, inhibition of miR-1247-5p could alleviate the induction of RANKL on osteoclast differentiation. *n* = 4, **P < 0.01. **(H)** TRAP staining was accomplished in osteoclasts overexpressing/inhibiting miR-1247-5p in the presence of RANKL, M-CSF and TNF-α for 5 days. **(I)** Summarized data showed that miR-1247-5p promoted TNF-α-induced osteoclast differentiation. AMO-1247-5p attenuated the promoting effect of TNF-α on osteoclasts differentiation. *n* = 4, **P < 0.01.

To explore the molecular mechanisms by which circHmbox1 participated in TNF-α-induced osteoclasts differentiation, we firstly used the algorithms of circBase to predict the putative targeting miRNAs of circHmbox1. Some miRNAs were predicted to bind to circHmbox1, including miR-1247-5p, miR-17-3p, miR-200c-5p, miR-222-3p, miR-27a-3p, miR-541-5p, miR-666-3p and miR-690. We then tested these miRNAs expression by qRT-PCR in TNF-α-induced osteoclasts differentiation. The results showed that the expressions of miR-1247-5p, miR-222-3p and miR-666-3p were increased in RANKL-induced osteoclastogenesis (Figure 4A). Among these microRNAs, miR-1247-5p expression was further increased in TNF-α-induced osteoclasts differentiation, which was corresponded to the regulation of circHmbox1 by TNF-α in osteoclasts. Complementation between circHmbox1 and “seed sequence” of miR-1247-5p were showed in Figure 4B with yellow highlights. Dual luciferase reporter assay further showed a significant decrease in the firefly luciferase activity when pMIR-circHmbox1 was cotransfected with miR-1247-5p mimics, suggesting that miR-1247-5p may be a high-affinitive target of circHmbox1 in osteoclasts (Figure 4C).

To further confirm whether circHmbox1 could bind to miR-1247-5p in osteoclasts, we tested the expression of miR-1247-5p in osteoclasts overexpressing or silencing circHmbox1. We found that the level of miR-1247-5p was higher in the osteoclasts transfected with si-circHmbox1 compared with the cells transfected with si-NC. However, miR-1247-5p expression was significantly inhibited in the osteoclasts overexpressing circHmbox1 (Figure 4D). In addition, the expression of miR-1247-5p was increased in TNF-α-induced osteoclasts differentiation, which was alleviated by overexpressing circHmbox1 in osteoclasts. Silencing of circHmbox1 could further enhance the promoting effect of TNF-α on miR-1247-5p expression in osteoclasts, suggesting that circHmbox1 was involved in TNF-α-increased miR-1247-5p expression in osteoclasts (Figure 4E).

To further examine whether miR-1247-5p was involved in osteoclasts differentiation, miR-1247-5p was overexpressed or silenced in pre-osteoclasts with miR-1247-5p mimic and AMO-1247-5p respectively, and then the cells were induced with RANKL and M-CSF for 7 days. TRAP staining showed that miR-1247-5p could promote RANKL-induced osteoclast differentiation. However, inhibition of miR-1247-5p could decrease the induction of osteoclasts differentiation by RANKL (Figures 4F,G). We also tested the effect of miR-1247-5p on TNF-α-induced osteoclasts differentiation. The results of TRAP staining showed that miR-1247-5p promoted TNF-α-induced osteoclast differentiation. AMO-1247-5p inhibited the promoting effect of TNF-α on osteoclasts differentiation (Figures 4H,I). Taken together, these results confirmed that miR-1247-5p targeted by circHmbox1 is involved in TNF-α-induced osteoclasts differentiation.

### MiR-1247-5p Targeted by CircHmbox1 Is Involved in TNF-α-Induced Osteoclasts Differentiation

**FIGURE 5:**
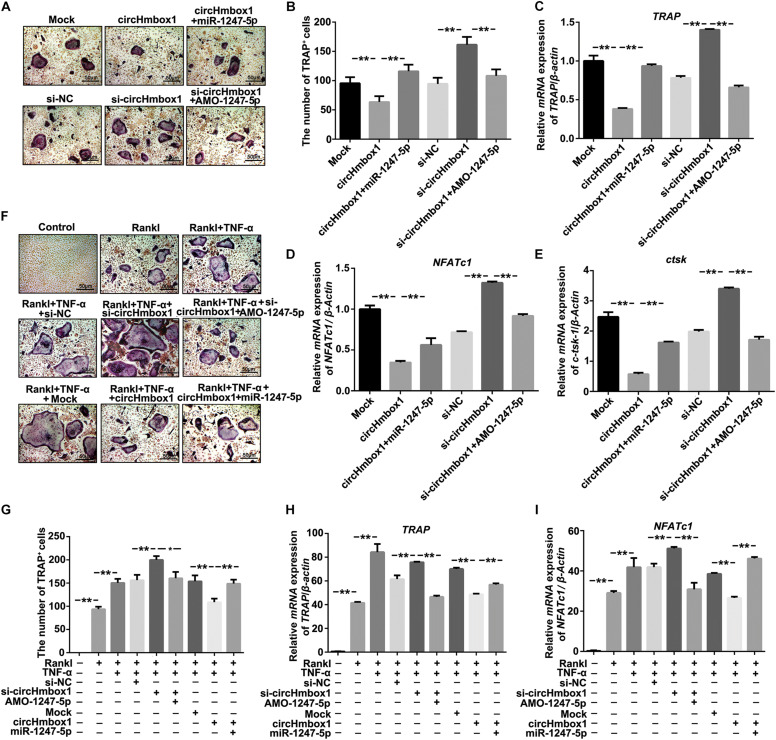
MiR-1247-5p targeted by circHmbox1 is involved in TNF-α-induced osteoclasts differentiation. **(A)** TRAP staining was showed in osteoclasts transfected with circHmbox1/si-circHmbox1 and miR-1247-5p/AMO-1247 in the presence of RANKL and M-CSF for 5 days. **(B)** Summarized data showed that miR-1247-5p attenuated circHmbox1-inhibited osteoclasts differentiation. However, promoting of osteoclasts differentiation by si-circHmbox1 was alleviated by AMO-1247-5p. *n* = 6, **P < 0.01. **(C–E)** The expressions of *TRAP* **(C)**, *NFATc1***(D)** and *ctsk* **(E)** were analyzed by qRT-PCR in osteoclasts transfected with circHmbox1/si-circHmbox1 and miR-1247-5p/AMO-1247 in the presence of RANKL and M-CSF for 5 days. *n* = 3, **P < 0.01. **(F)** TRAP staining was showed in osteoclasts transfected with circHmbox1/si-circHmbox1 and miR-1247-5p/AMO-1247 in the presence of RANKL, M-CSF and TNF-α for 5 days. **(G)** Summarized data showed that silencing of circHmbox1 could enhance TNF-α-induced osteoclast differentiation, which could be reduced by AMO-1247-5p. MiR-1247-5p also weakened the inhibitory effect of circHmbox1 on TNF-α-induced osteoclast differentiation. *n* = 6, *P < 0.05, **P < 0.01. **(H,I)** The expressions of *TRAP* **(H)** and *NFATc1***(I)** were analyzed by qRT-PCR in osteoclasts transfected with circHmbox1/si-circHmbox1 and miR-1247-5p/AMO-1247 in the presence of RANKL, M-CSF and TNF-α for 5 days. *n* = 3, **P < 0.01.

To examine whether miR-1247-5p targeted by circHmbox1 was involved in TNF-α-induced osteoclasts differentiation, we changed the expressions of miR-1247-5p with miR-1247-5p mimic or AMO-1247-5p in osteoclasts overexpressing or silencing circHmbox1. TRAP staining showed that miR-1247-5p attenuated circHmbox1-inhibited osteoclasts differentiation. However, increasing of osteoclasts differentiation by si-circHmbox1 was alleviated by AMO-1247-5p (Figures 5A,B). QRT-PCR analysis revealed that the downregulation of osteoclast-specific genes (*TRAP*, *NFATc1* and *ctsk*) expression by circHmbox1 were significantly alleviated by miR-1247-5p in osteoclasts. AMO-1247-5p also prevented these genes upregulation in osteoclasts transfected with si-circHmbox1, suggesting that miR-1247-5p was involved in the regulation of osteoclasts differentiation by circHmbox1 (Figures 5C–E). In addition, we found that silencing of circHmbox1 could enhance the promoting effect of TNF-α on osteoclast differentiation, which could be alleviated by AMO-1247-5p. MiR-1247-5p also weakened the inhibitory effect of circHmbox1 on osteoclast differentiation induced by TNF-α (Figures 5F,G). Similar results were further demonstrated by the measurements of osteoclast-specific genes expression (Figures 5H,I). These results concluded that TNF-α stimulated osteoclasts differentiation primarily through circHmbox1 binding to miR-1247-5p.

### Exosomal With Low CircHmbox1 Expression From Osteoclasts Treated With TNF-α Inhibits Osteoblast Differentiation Primarily Through Targeting miR-1247-5p

**FIGURE 6:**
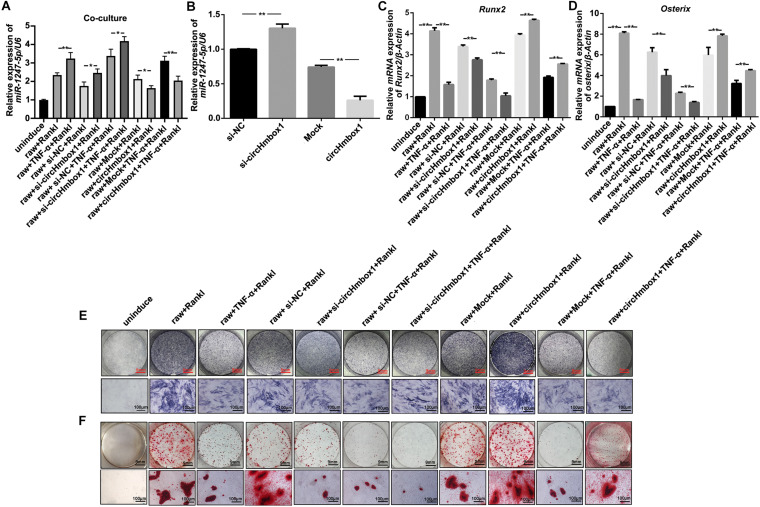
Exosomal with low circHmbox1 expression from osteoclasts treated with TNF-α inhibits osteoblast differentiation primarily through targeting miR-1247-5p. **(A)** The expressions of miR-1247-5p were analyzed by qRT-PCR in osteoblasts co-cultured with Raw264.7 cells transfected with circHmbox1/si-circHmbox1 in the presence of RANKL and TNF-α. n = 3, **P < 0.01. **(B)** The expressions of miR-1247-5p were analyzed by qRT-PCR in osteoblasts transfected with circHmbox1/si-circHmbox1. n = 3, **P < 0.01. **(C,D)** The expressions of *Runx2* **(C)** and *Osterix* **(D)** were analyzed by qRT-PCR in osteoblasts co-cultured with Raw264.7 cells transfected with circHmbox1/si-circHmbox1 in the presence of RANKL and TNF-α. n = 3, **P < 0.01. **(E,F)**: ALP staining **(E)** and AR staining **(F)** were observed in osteoblasts co-cultured with Raw264.7 cells transfected with circHmbox1/si-circHmbox1 in the presence of RANKL and TNF-α. *n = 3.*

To further test whether exosomal with low circHmbox1 expression transferred from osteoclasts could affect miR-1247-5p expression in osteoblasts, we co-cultured osteoclasts with osteoblasts in a transwell system with a 0.4-μm pore. QRT-PCR results showed that the level of miR-1247-5p in osteoblasts co-cultured with Raw264.7 cells treated with RANKL and TNF-α was obviously higher than that in osteoblasts co-cultured with RANKL-induced Raw264.7 cells (Figure 6A). The expression of miR-1247-5p was increased in osteoblasts co-cultured with osteoclasts transfected with si-circHmbox1 when compared with those in osteoblasts co-cultured with osteoclasts transfected with si-NC. However, miR-1247-5p expression was decreased in osteoblasts co-cultured with osteoclasts transfected with circHmbox1, suggesting that exosomal circHmbox1 from osteoclasts could regulate miR-1247-5p expression in osteoblasts (Figure 6A). Furthermore, the level of miR-1247-5p expression was increased in osteoblasts co-cultured with osteoclasts transfected with si-circHmbox1 compared to si-NC transfection in the presence of TNF-α. MiR-1247-5p expression was decreased in osteoblasts co-cultured with osteoclasts transfected circHmbox1 compared to mock transfection in the presence of TNF-α, suggesting that TNF-α might affect miR-1247-5p expression in osteoblasts primarily through regulating osteoclasts to secrete exosomes circHmbox1(Figure 6A).

To further confirm the regulation of miR-1247-5p expression by circHmbox1 in osteoblasts, we increased and decreased the expressions of circHmbox1 in osteoblasts, and then tested the expression of miR-1247-5p by qRT-PCR. The results showed that si-circHmbox1 could increase the expression of miR-1247-5p, whereas overexpression of circHmbox1 reduced miR-1247-5p expression in osteoblasts (Figure 6B), indicating that circHmbox1 regulated miR-1247-5p expression in osteoblasts.

Osteoblasts co-cultured with osteoclasts were used to test whether exosomes circHmbox1 from osteoclasts could affect osteoblasts differentiation. The results showed that the expressions of osteoblast marker genes (*Runx2* and *Osterix*) were decreased in osteoblasts co-cultured with osteoclasts transfected with si-circHmbox1 when compared with those in osteoblasts co-cultured with osteoclasts transfected with si-NC. However, *Runx2* and *Osterix* expressions were increased in osteoblasts co-cultured with osteoclasts transfected circHmbox1, suggesting that exosomal circHmbox1 from osteoclasts could regulate osteoblasts differentiation (Figures 6C,D). To further confirm whether TNF-α could inhibit osteoblasts differentiation through regulating osteoclasts to secrete exosomes with low circHmbox1 expression, osteoblasts were co-cultured with osteoclasts overexpressing or silencing circHmbox1 in the presence of TNF-α. The results of qRT-PCR showed that the osteoblast marker genes (*Runx2* and *Osterix*) were decreased in osteoblasts co-cultured with osteoclasts treated with TNF-α, which was further inhibited in osteoblasts co-cultured with osteoclasts silencing circHmbox1 under TNF-α. Osteoblasts differentiation was enhanced in osteoblasts co-cultured with osteoclasts overexpressed circHmbox1 under TNF-α (Figures 6C,D). Similar results were observed with ALP staining and AR staining (Figures 6E,F). These results indicated that TNF-α might be inhibit osteoblasts differentiation primarily through promoting osteoclasts to secrete exosomes with low circHmbox1 expression.

### Molecular Targets of miR-1247-5p Are Involved in TNF-α-Regulated Osteoclasts Differentiation and Osteoblastogenesis

**FIGURE 7:**
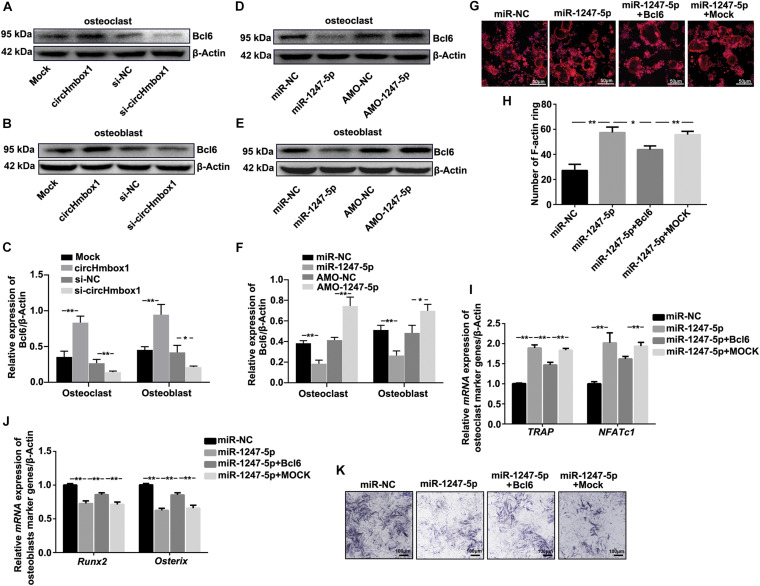
Molecular targets of miR-1247-5p are involved in TNF-α-regulated osteoclasts differentiation and osteoblastogenesis. **(A–C)** The expressions of Bcl6 were analyzed by western blot in osteoclasts **(A)** or osteoblasts **(B)** transfected with circHmbox1 or si-circHmbox1. *n = 3*, **P < 0.01, *P < 0.05. **(D–F)** The expressions of Bcl6 were analyzed by western blot in osteoclasts **(D)** or osteoblasts **(E)** transfected with miR-1247-5p or AMO-1247-5p. *n* = 3, **P < 0.01, *P < 0.05. **(G,H)** F-actin ring staining showed that miR-1247-5p significantly increased osteoclast differentiation, whereas overexpressing of Bcl6 obviously alleviated the promoting effect of miR-1247-5p on osteoclast differentiation. **(I)** The expressions of *TRAP* and *NFATc1* were analyzed by qRT-PCR in pre-osteoclasts transfected with miR-1247-5p and/or Bcl6 under RANKL and M-CSF for 5 days. *n* = 3, **P < 0.01. **(J)** The expressions of *Runx2* and Osterix were analyzed by qRT-PCR in osteoblasts transfected with miR-1247-5p and/or Bcl6 under osteogenic induction medium for 7 days. *n* = 3, **P < 0.01. **(K)** Osteoblasts transfected with miR-1247-5p and/or Bcl6 were cultured in osteogenic induction medium for 7 days, followed by ALP staining. *n = 4.*

Based on the above results, miR-1247-5p was confirmed to involve in TNF-α-regulated osteoclastogenesis and osteoblastogenesis. Thus, miR-1247-5p might be target several factors to regulate osteoclastogenesis and osteoblastogenesis. A computation and bioinformatics-based approach was first used to predict the putative targets of miR-1247-5p. These explorations lead to the identification of candidate targets of miR-1247-5p: Bcl6. The results of western blot showed that the expression of Bcl6 was upregulated by overexpressing of circHmbox1 in osteoclasts and in osteoblasts, which was decreased by silencing of circHmbox1 (Figures 7A–C). Furthermore, inhibition of miR-1247-5p obviously increased the expression of Bcl6 in osteoclasts and in osteoblasts. However, the levers of Bcl6 expression were downregulated by miR-1247-5p in osteoclasts and osteoblasts (Figures 7D–F). To further test whether Bcl6 was involved in the regulation of the differentiation of osteoblasts and osteoclasts by miR-1247-5p, we decreased and increased the expressions of Bcl6 in osteoclasts and osteoblasts, respectively. F-actin ring staining and qRT-PCR analysis of osteoclast-specific genes (*TRAP* and *NFATc1*) showed that miR-1247-5p increased osteoclast differentiation, whereas overexpressing of Bcl6 obviously alleviated the promoting effect of miR-1247-5p on osteoclast differentiation (Figures 7G–I). In addition, qRT-PCR analysis showed that osteoblast-specific genes (*Runx2* and *Osterix*) were lower in the osteoblasts overexpressing miR-1247-5p compared with the cells transfected with the miR-NC, whereas overexpression of Bcl6 obviously attenuated the inhibition of these osteoblast-specific genes expression by miR-1247-5p (Figure 7J). Similar results were observed by ALP staining (Figure 7K), suggesting that Bcl6 was involved in miR-1247-5p-inhibited osteoblast differentiation.

### Overexpression of CircHmbox1 *in vivo* Alleviates OVX-Induced Osteoporosis

**FIGURE 8:**
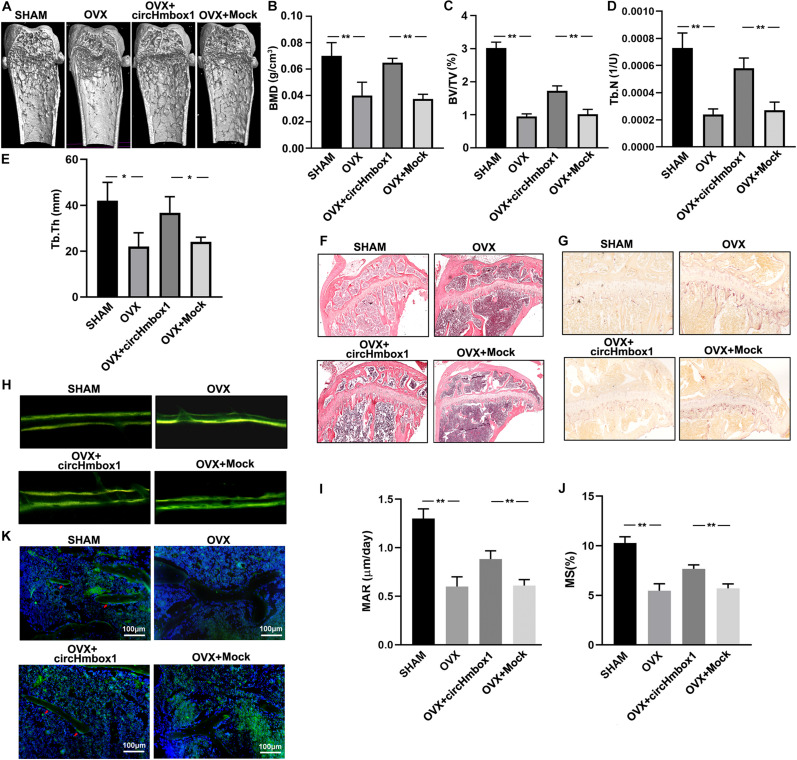
Overexpression of circHmbox1 *in vivo* alleviates OVX-induced osteoporosis. **(A)** Representative figures of micro-CT analysis of the distal end of intact femurs of SHAM mice, OVX mice, OVX+ circHmbox1 mice and OVX+Mock mice. **(B–E)** BMD, BV/TV, Tb.N and Tb.Th in the region of interest were measured. *n = 4* mice per group, **P < 0.01, *P < 0.05. **(F)** HE staining was performed to histologically identify structures of the distal end of intact tibias of SHAM mice, OVX mice, OVX+ circHmbox1 mice and OVX+Mock mice. **(G)** TRAP staining was showed in the metaphyseal area of tibias bone sections derived from different groups. *n = 4*. **(H–J)** Dynamic bone formation parameters were assessed by repeated injection of tetracycline, respectively nine and one day before sacrifice. The MAR **(I)** and MS **(J)** were comparable. *n* = 4, **P < 0.01. **(K)** The immunofluorescence staining showed that circHmbox1 alleviated OVX-inhibited osteoblastic marker osteopontin expression (green fluorescence) in the bone trabecula surface. *n = 4*.

OVX mice were injected with circHmbox1-CH to investigate the effect of circHmbox1 on OVX-induced osteoporosis *in vivo*. Micro-CT analysis revealed that the BMD, BV/TV, Tb.N and Tb.Th in OVX mice were significantly decreased compared to SHAM mice. However, injection of circHmbox1-CH significantly improved the trabecular bone mass and microarchitecture in OVX mice (Figures 8A–E), in consistence with the less amount of trabecular bones shown in HE staining (Figure 8F). TRAP staining of the tibias bone sections showed that the number of osteoclasts and surface area were obviously increased in OVX mice, which were decreased in OVX mice injected with circHmbox1-CH (Figure 8G).

To further investigate the effect of circHmbox1 on bone formation *in vivo*, dynamic bone formation parameters of mice were measured by repeated injection of tetracycline, respectively, nine and one day before sacrifice (Figure 8H). The mineral apposition rate (MAR) and mineralizing surface (MS) was comparable between groups to estimate normal osteoblast function and number. The MAR and MS were increased in circHmbox1-CH treated OVX mice compared with Mock treated OVX mice (Figures 8I,J), suggesting that circHmbox1 could increase bone formation *in vivo*. In addition, The immunofluorescence staining showed that circHmbox1 alleviated OVX-inhibited osteoblastic marker osteopontin expression in the bone trabecula surface, which was evidenced by enhanced green fluorescence of osteopontin in circHmbox1-CH-treated OVX mice compared with OVX mice (Figure 8K).

## Discussion

Estrogen hormone decline in postmenopausal women lead to the increase of spontaneous proinflammatory TNF-α production, which is a critical risk factor for the development of PMOP. Many studies have confirmed that TNF-α could enhance the ability of osteoclasts to absorb bone and reduce the ability of osteoblasts to form bone (David and Schett, 2010). However, it was unclear whether circRNA was involved in the regulation of osteoblasts differentiation and osteoclasts differentiation by TNF-α. Results of the present studies for the first time demonstrated that the level of circHmbox1 expression was significantly decreased in TNF-α-induced osteoclasts differentiation. CircHmbox1 was involved in TNF-α-induced osteoclasts differentiation primarily through sponging miR-1247-5p to regulate Bcl6 expression. In addition, TNF-α might inhibit osteoblasts differentiation through promoting osteoclasts to secrete exosomes with low circHmbox1 expression. Exosomal with low circHmbox1 expression transferred from osteoclasts decreased circHmbox1 expression in osteoblasts and inhibited osteoblasts differentiation through regulating miR-1247-5p/Bcl6. Taken together, these results indicated that circHmbox1-targeting miR-1247-5p was involved in the regulation of bone metabolisms by TNF-α in PMOP.

Many studies have confirmed that circRNAs played crucial roles in cells differentiation. Du *et al.* demonstrated that circFgfr2 was a potential new target for bone formation, which promoted osteoblasts differentiation through regulating miR-133/BMP6 (Du et al., 2019). Li et al. found that circular RNA CDR1 could regulate osteoblastic differentiation via the miR-7/GDF5/SMAD and p38 MAPK signaling pathway (Li et al., 2018). In addition, circRNAs was confirmed to involve in the development of inflammatory disease. Fang et al. found that circANKRD36 was associated with inflammation in patients with type 2 diabetes mellitus (Fang et al., 2018). Hsa_circ_0045714 could regulate chondrocyte to synthesize extracellular matrix through miR-193b targeting IGF1R in osteoarthritis (Li et al., 2017). However, no studies were found to test the roles of circRNAs in the regulation of bone metabolisms by TNF-α in PMOP. The present study for the first time screened the expression of circRNAs-associated osteoclastogenesis in TNF-α-inhibited osteoclasts differentiation and demonstrated that circHmbox1 played important roles in the regulation of osteoclasts differentiation and osteoblasts differentiation by TNF-α. However, the exact mechanisms by which TNF-α regulate circHmbox1 expression in osteoclasts need to be further explored.

Growing evidences suggested that circRNAs could harbor miRNA binding sites and usually acted as miRNA sponges to perform biological functions (Haddad and Lorenzen, 2019). In the present study, we found that some miRNAs were predicted to bind to circHmbox1, in which only miR-1247-5p was examined as target of circHmbox1 to involve in TNF-α-induced osteoclasts differentiation. Previous studies confirmed that miR-1247-5p was associated with the development of many diseases, such as cancer and bone metabolic disease. MiR-1247-5p was also confirmed as targets of many circRNAs to regulate cells biological functions. Besides serving as miRNA sponges, circRNAs several functions have been identified, including transcriptional activators (Xie et al., 2017) or even translating into proteins (Fang et al., 2018). However, it was unclear whether circHmbox1 had other roles in TNF-α-induced osteoclasts differentiation. The other functions of circHmbox1 in TNF-α-induced osteoclasts differentiation will be tested in the future studies.

Exosomes, carrying messenger RNAs (mRNAs), miRNAs, and double-stranded DNA, could merge with and release their contents into cells that are distant from their cell of origin, which was recognized as an important way for exosomes to mediate cell-to-cell signaling and influence physiology processes of cells (Wang et al., 2019). CircRNAs were stable in exosomes and could be transferred in to exosomes from many cells. Many studies confirmed that exosomal circRNAs-mediated intercellular communication was involved in the pathogenesis of disease. Liu *et al.* found that exosomal circRNA_100284 from arsenite-transformed cells could promote cell proliferation through miR-217 targeting EZH2 in the malignant transformation of human hepatic cells (Dai et al., 2018). Exosome circRNA from adipocytes accelerated the growth of hepatocellular carcinoma via targeting deubiquitination-related USP7 (Zhang et al., 2019). Furthermore, some exosomal miRNAs were demonstrated to play important roles in the interaction between osteoblasts and osteoclasts (Li et al., 2016). However, up to now, no studies investigated the role of exosome circRNA from bone cells in the bone metabolic disease. Our results for the first time showed involvement of exosome circRNA in the interaction between osteoblasts and osteoclasts.

Bcl6 is a transcriptional repressor that is essential to control physiological osteoclast development and maintenance of bone homeostasis. Miyauchi et al. found that Bcl6-deficient mice increased osteoclast differentiation and reduced bone mass. Further studies showed that Bcl6 inhibited osteoclasts differentiation by inhibiting osteoclastic transcription genes, such as *NFATc1* (Miyauchi et al., 2010). In contrast, Bcl6-deficient mice exhibited significant attenuated osteoblast differentiation. Atsuhiro *et al.* confirmed that Bcl6 was expressed in osteoblasts and demonstrated that it promoted osteoblastogenesis by inhibiting Stat1 (Fujie et al., 2015). These studies implicated that Bcl6 play opposite roles in osteoblasts differentiation and osteoclasts differentiation. In the present study, we found that Bcl6 was functional target gene for miR-1247-5p-mediated osteoblasts differentiation and osteoclasts differentiation. Bcl6 play negative roles in the regulation of osteoblasts differentiation and osteoclasts differentiation.

Our results showed that injection of circHmbox1-CH significantly improved the trabecular bone structures in OVX mice, indicating that increasing the level of circHmbox1 might be effective for the treatment of OVX-induced osteoporosis. Gene therapy targeting miRNAs might be a feasible and effective method to alleviate the progression of the disease. However, the major challenge of ribonucleotide-based therapeutics is the rapid degradation of nucleic acids *in vivo*. Compared to linear miRNA inhibitor administration, circRNAs can resist against nuclease degradation and have the potential to sustain miRNA suppression for prolonged time periods and would in turn require minimal dosage, or longer dosing intervals. Furthermore, the majority of circRNAs are conserved across species and often exhibit tissue specific expression. Thus, circRNA-based therapy may be more specific than siRNA-based therapy.

In conclusion, our data provided new evidence that circHmbox1-targeting miR-1247-5p/Bcl6 was involved in the regulation of osteoclasts differentiation and osteoblasts differentiation by TNF-α. This study is an effort to establish a molecular mechanism of TNF-α-regulated bone metabolisms in PMOP, and to provide insights into the potential contribution of circRNA in the regulation of osteoclasts differentiation and osteoblasts differentiation by TNF-α.

## Data Availability Statement

The raw data supporting the conclusions of this article will be made available by the authors, without undue reservation.

## Ethics Statement

The animal study was reviewed and approved by Shanghai Jiao Tong University Animal Study Committee.

## Author Contributions

All authors contributed to the design of the study. LG and FH analyzed data. ZL, CL, XX, BL, and MJ performed research. LG and TY collected the data and performed the analysis. LG, PH, LD, and TY wrote the manuscript. All authors interpreted the findings, revised the manuscript, and approved the final version. LG and TY takes responsibility for the integrity of this work.

## Conflict of Interest

The authors declare that the research was conducted in the absence of any commercial or financial relationships that could be construed as a potential conflict of interest.

## Abbreviations

α-MEM: alpha-minimal essential media
3′-UTR: 3′-untranslational region
ALP: alkaline phosphatase
AMO: anti-microRNA oligonucleotides
ARS: alizarin red S
Bcl6: B cell lymphoma 6
BMD: bone mineral density
BMMs: bone marrow macrophages cells
BV/TV: bone volume against tissue volume
CH: chitosan
circRNAs: circular RNAs
DIO, 3: 3′-dioctadecyloxacarbocyanine perchlorate
FBS: fetal bovine serum
HE: hematoxylin eosin
MAR: mineral apposition rate
M-CSF: macrophage-colony stimulating factor
micro-CT: micro computed tomography
miRNAs: microRNAs
mRNAs: messenger RNAs
MS: mineralizing surface
NC: negative control
OVX: ovariectomized
PET: pore polyethylene terephthalate
PMOP: postmenopausal osteoporosis
RANKL: receptor activator of nuclear factorκ-B ligand
siRNAs: small interfering RNAs
Tb.N: trabecular number
Tb.Sp: trabecular Separation/Spacing
TNF-α: tumor necrosis factor-alpha
TRAP: tartrate resistant acid phosphatase.
